# Supplementary material for: Compressive Alginate Sponge Derived from Seaweed Biomass Resources for Methylene Blue Removal from Wastewater
Source: Polymers (Basel). 2019 Jun 2;11(6):961. doi: 10.3390/polym11060961 (PMC6631128; doi:10.3390/polym11060961)
Supplement: Supplementary file 1 [file polymers-11-00961-s001.pdf]

Electronic Supplementary Information

Compressive alginate sponge derived from seaweed biomass resources for methylene blue removal from wastewater

Xiaojun Shen[1,2], Panli Huang[1], Fengfeng Li[1], Xiluan Wang[1*], Tongqi Yuan[1*], Runcang Sun[1,3]

1 Beijing Key Laboratory of Lignocellulosic Chemistry, Beijing Forestry University, Beijing, 100083, China; shenxiaojun@iccas.ac.cn (X.S.); huangpanli@bjfu.edu.cn (P.H.); qhxylifeng@163.com (F.L.); wangxiluan@bjfu.edu.cn (X.W.); ytq581234@bjfu.edu.cn (T.Y.); rcsun3@bjfu.edu.cn (R.S.)

2 Beijing National Laboratory for Molecular Sciences, Key Laboratory of Colloid and Interface and Thermodynamics, Institute of Chemistry, Chinese Academy of Sciences, Beijing 100190, China; shenxiaojun@iccas.ac.cn (X.S.)

3 Center for Lignocellulose Science and Engineering, and Liaoning Key Laboratory Pulp and Paper Engineering, Dalian Polytechnic University, Dalian, China; rcsun3@bjfu.edu.cn (R.S.); rcsun3@bjfu.edu.cn (R.S.)

* Corresponding authors. Address: wangxiluan@bjfu.edu.cn and ytq581234@bjfu.edu.cn; Tel: +86-10-62336903

Supplementary detailed physical properties of AS

Typically, sodium alginate was extracted from seaweed according to our previous work [43] and it was highly soluble in water to obtain aqueous solution. After the first-step-lyophilization at −50 ^{o}C for 48 h, a freestanding while fragile lyophilized sample was obtained initially. Once thoroughly immersed the lyophilized sample in 5 wt % $CaCl_2$ aqueous solution for 12 h, the Ca^{2+} ions were diffused and penetrated into the network of the lyophilized sample through ionic reaction. After then, the second-step-lyophilization was processed to obtain a final AS. Even after mechanical cutting by scissors or knife, the ASs in different shapes still maintained its original elasticity. Furthermore, 3D multiporosity is an important factor for uptaking adsorbate with accessible active site, enhancing adsorption rate and allowing fast adsorbate diffusion [S1, S2]. The formation of multi-porous structures of AS was mainly induced by the template of ice from solutions under lyphilization. During the two-step lyophilization processes, water from solutions were rapidly freezing and then transformed into vapor without passing through the liquid phase under high vacuum condition. The pores of AS nearly kept the shapes of ice crystals and resulting 3D porous sponge-like framework with a large specific surface area. Varying with the temperature and the moisture content during two-step lyophilization, numerous "riband bridges" were protruded from the pore walls and connect adjacent layers and divide long pores into small parts. As mentioned, the AS has a well-defined, interconnected 3D porous network and large specific surface area, which was superior to conventional compacted alginate-based materials [S1-S4]. Generally, 3D porous sponge network is beneficial to pollutant removal by increasing the contact frequency between the

pollutants and adsorbents [S2]. Moreover, the adsorption performance of adsorbents is also highly dependent on the internal porous structure [S5]. The macroporosities promote the accessibility to active surface, in the meantime, the micro- and mesoporosities provide large active surfaces, both were advantageous to pollutant entrapment.

Supplementary detailed chemical properties of AS

The chemical composition of AS prepared via the green two-step lyophilization method were characterized by FT-IR analysis, its typical peaks were assigned according to previous publications [S6-S8]. The FT-IR spectrum of AS exhibited a strong absorption band at 3386 cm^{-1}, which was associated with the abundant –OH groups existed in alginate. The adsorption band at 2927 cm^{-1} was attributed to the aliphatic C–H vibrations of polysaccharide skeleton. The peaks observed at 1607 and 1422 cm^{-1} were contributed by the vibration of the C=O group, and the signals at 1038 cm^{-1} was due to the C–O–C group. Based on results of FT-IR analysis, the existence of a large number of functional groups, such as hydroxyl, carbonyl and carboxyl groups penetrated in the AS network can be deduced, similar to that of relevant literature [6-8]. It indicated that the structural composition of AS could be maintained during the two-step lyophilization process without structural defects. Simultaneously, the resultant XRD pattern exhibited none of distinct crystalline formation, consistent with the amorphous structure of alginate species [S1]. As shown in Fig. 2g, The C 1s spectrum of AS indicated typical carbon-linked peaks with binding energies which were differentiated via deconvolution. These peaks can be assigned to the carbon atoms binding in functional groups: C–C/C=C (284.6 eV), C–O (286.2 eV), C=O (288.0 eV) and O–C=O (288.9eV), respectively. In addition, the peaks at 347.5 and 350.9 eV in Fig. 2h were mainly resultant from Ca 2p3/2 and Ca 2p1/2, respectively, indicative of the existence of bivalent Ca^{2+} ions. During the crosslink process between the two-step lyophilization route, Ca^{2+} ions combined with oxygen-contained groups (e.g., –COO or –OH) on active surfaces of AS network and led to a cross-linking effect afterwards Na^{+} replaced by Ca^{2+}. Thus, the framework of the sponge adsorbent was strong enough and water-insoluble [S9, S10], ensuring free-standing stability and facile recyclability in waste water.

Extraction step

$$2\,M(Alg)_n + n\,NaCO_3 \longrightarrow 2n\,NaAlg + M_2(CO_3)_n$$

$$2\,NaAlg + CaCl_2 \longrightarrow Ca(Alg)_2\downarrow + 2\,NaCl$$

$$Ca(Alg)_2 + 2\,HCl \longrightarrow 2HAlg + CaCl_2$$

$$HAlg + NaOH \longrightarrow NaAlg + H_2O$$

Crosslink step

$$2NaAlg + CaCl_2 \longrightarrow Ca(Alg)_2\downarrow + 2\,NaCl$$

Scheme S1. The involved chemical equations of Scheme 1.

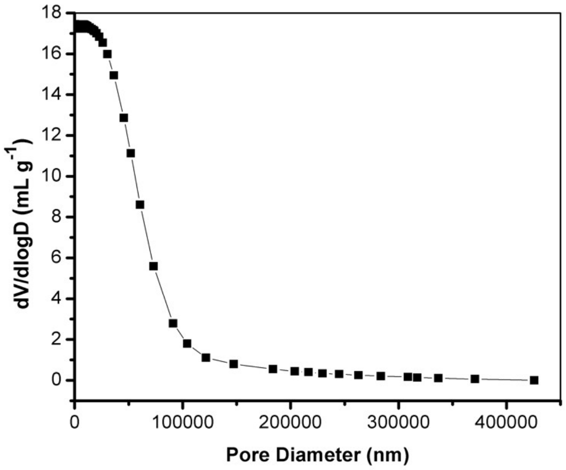

Figure S1. The pore size distributions of AS calculated by the Mercury Intrusion Porosimetry method.

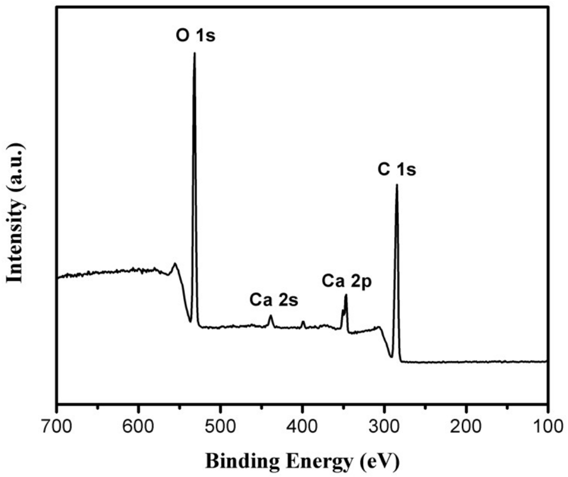

Figure S2. The XPS survey spectrum of the sample of AS.

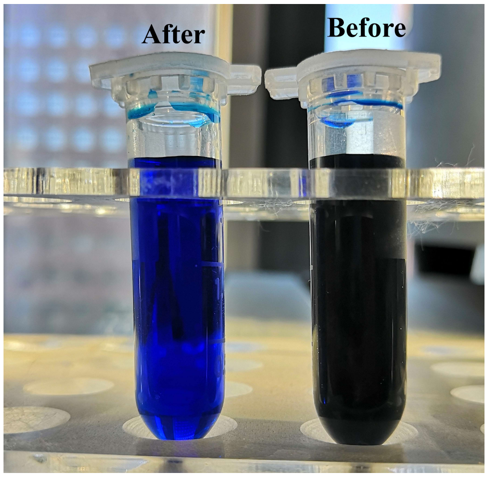

Figure S3. The images of MB solution before and after batch adsorption.

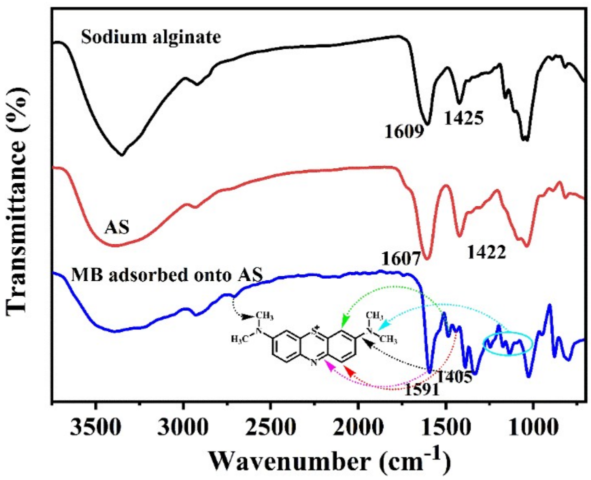

Figure S4. FT-IR spectra of sodium alginate, AS and MB adsorbed onto AS.

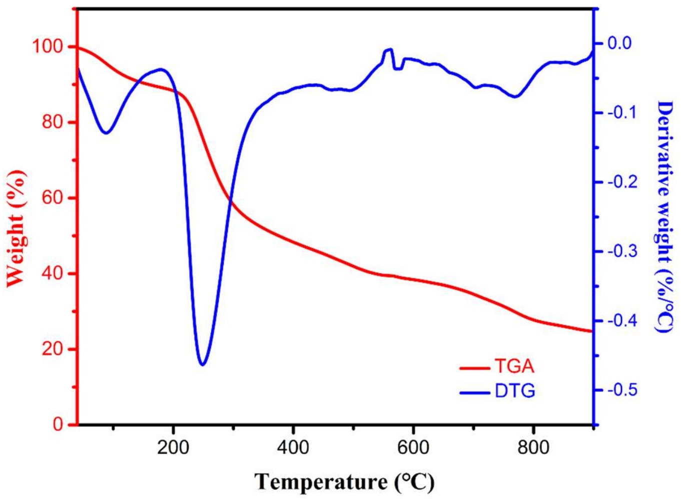

Figure S5. The TGA analysis result of the sample of AS.

Table S1. The MB adsorption capacity of alginate-based adsorbents in literatures.

**Adsorbent**	**Adsorption capacity (mg g^{-1})**	**Reference**
CNC-alginate hydrogel beads	73	S11
Graphene oxide/calcium alginate composites	182	S12
Alginate-halloysite nanotube beads	222	47
Alginate porous beads	278	S13
Sodium alginate (SA) beads	572	S14
Pure alginate hydrogel beads	600−650	S15
Calcium alginate beads	650	44
Spent coffee grounds into calcium-alginate	710	S16
Calcium alginate–bentonite–activated carbon	757	S17
GO-SA gel	799	46
NaAlg-*g*-p(AA-*co*-St) /I/S	1797	45
Alginate sponge	1279	This paper

Table S2. The MB adsorption capacity of other reported adsorbents in literatures.

**Adsorbent**	**Adsorption capacity (mg g^{-1})**	**Reference**
Activated carbon	36.25	25
Zeolites	31.60	29
Mesoporous silica	14.5	26
Magnetic Cellulose/Graphene Oxide Composite hybrid xerogel	70.03	61
Nanoporous alumina	427	28
Carbon-based nanomaterials	714.29	13

Plant fibre	131.6	36
Silk cotton	555.56	37
Leaves	285.7	38
Citrus waste peels	333.33	39
Bagasse	564	40
Rice husk	143.3	41
Chitosan	142	42
Guar gum	61.92	43
Alginate sponge	1279	This paper

Supporting References

S1. Deze, E.G.; Papageorgiou, S.K.; Favvas, E.P.; Katsaros, F.K. Porous alginate aerogel beads for effective and rapid heavy metal sorption from aqueous solutions: Effect of porosity in Cu^{2+} and Cd^{2+} ion sorption. *Chemical Engineering Journal* **2012**, *209*, 537-546, doi:https://doi.org/10.1016/j.cej.2012.07.133.

S2. Wang, L.; Cheng, C.; Tapas, S.; Lei, J.; Matsuoka, M.; Zhang, J.; Zhang, F. Carbon dots modified mesoporous organosilica as an adsorbent for the removal of 2,4-dichlorophenol and heavy metal ions. *Journal of Materials Chemistry A* **2015**, *3*, 13357-13364, doi:10.1039/C5TA01652E.

S3. Liu, G.; Hu, Z.; Guan, R.; Zhao, Y.; Zhang, H.; Zhang, B. Efficient removal of methylene blue in aqueous solution by freeze-dried calcium alginate beads. *Korean Journal of Chemical Engineering* **2016**, *33*, 3141-3148, doi:10.1007/s11814-016-0177-4.

S4. Lagoa, R.; Rodrigues, J.R. Kinetic analysis of metal uptake by dry and gel alginate particles. *Biochemical Engineering Journal* **2009**, *46*, 320-326, doi:https://doi.org/10.1016/j.bej.2009.06.007.

S5. Kosuge, K.; Kubo, S.; Kikukawa, N.; Takemori, M. Effect of Pore Structure in Mesoporous Silicas on VOC Dynamic Adsorption/Desorption Performance. *Langmuir* **2007**, *23*, 3095-3102, doi:10.1021/la062616t.

S6. Shamshina, J.L.; Gurau, G.; Block, L.E.; Hansen, L.K.; Dingee, C.; Walters, A.; Rogers, R.D. Chitin–calcium alginate composite fibers for wound care dressings spun from ionic liquid solution. *Journal of Materials Chemistry B* **2014**, *2*, 3924-3936, doi:10.1039/C4TB00329B.

S7. Zhang, H.; Lv, X.; Zhang, X.; Wang, H.; Deng, H.; Li, Y.; Xu, X.; Huang, R.; Li, X. Antibacterial and hemostatic performance of chitosan–organic rectorite/alginate composite sponge. *RSC Advances* **2015**, *5*, 50523-50531, doi:10.1039/C5RA08569A.

S8. Hassan, A.F.; Abdel-Mohsen, A.M.; Fouda, M.M.G. Comparative study of calcium alginate, activated carbon, and their composite beads on methylene blue adsorption. *Carbohydrate Polymers* **2014**, *102*, 192-198, doi:https://doi.org/10.1016/j.carbpol.2013.10.104.

S9. Jiao, C.; Xiong, J.; Tao, J.; Xu, S.; Zhang, D.; Lin, H.; Chen, Y. Sodium alginate/graphene oxide aerogel with enhanced strength–toughness and its heavy metal adsorption study. *International Journal of Biological Macromolecules* **2016**, *83*, 133-141, doi:https://doi.org/10.1016/j.ijbiomac.2015.11.061.

S10. Fei, Y.; Li, Y.; Han, S.; Ma, J. Adsorptive removal of ciprofloxacin by sodium alginate/graphene oxide composite beads from aqueous solution. *Journal of Colloid and Interface Science* **2016**, *484*, 196-204, doi:https://doi.org/10.1016/j.jcis.2016.08.068.

S11. Mohammed, N.; Grishkewich, N.; Berry, R.M.; Tam, K.C. Cellulose nanocrystal–alginate hydrogel beads as novel adsorbents for organic dyes in aqueous solutions. *Cellulose* **2015**, *22*, 3725-3738, doi:10.1007/s10570-015-0747-3.

S12. Li, Y.; Du, Q.; Liu, T.; Sun, J.; Wang, Y.; Wu, S.; Wang, Z.; Xia, Y.; Xia, L. Methylene blue adsorption on graphene oxide/calcium alginate composites. *Carbohydrate Polymers* **2013**, *95*, 501-507, doi:https://doi.org/10.1016/j.carbpol.2013.01.094.

S13. Peretz, S.; Anghel, D.F.; Vasilescu, E.; Florea-Spiroiu, M.; Stoian, C.; Zgherea, G. Synthesis, characterization and adsorption properties of alginate porous beads. *Polymer Bulletin* **2015**, *72*, 3169-3182, doi:10.1007/s00289-015-1459-4.

S14. Lu, T.; Xiang, T.; Huang, X.-L.; Li, C.; Zhao, W.-F.; Zhang, Q.; Zhao, C.-S. Post-crosslinking towards stimuli-responsive sodium alginate beads for the removal of dye and heavy metals. *Carbohydrate Polymers* **2015**, *133*, 587-595, doi:https://doi.org/10.1016/j.carbpol.2015.07.048.

S15. Jeon, Y.S.; Lei, J.; Kim, J.-H. Dye adsorption characteristics of alginate/polyaspartate hydrogels. *Journal of Industrial and Engineering Chemistry* **2008**, *14*, 726-731, doi:https://doi.org/10.1016/j.jiec.2008.07.007.

S16. Jung, K.-W.; Choi, B.H.; Hwang, M.-J.; Jeong, T.-U.; Ahn, K.-H. Fabrication of granular activated carbons derived from spent coffee grounds by entrapment in calcium alginate beads for adsorption of acid orange 7 and methylene blue. *Bioresource Technology* **2016**, *219*, 185-195, doi:https://doi.org/10.1016/j.biortech.2016.07.098.

S17. Benhouria, A.; Islam, M.A.; Zaghouane-Boudiaf, H.; Boutahala, M.; Hameed, B.H. Calcium alginate–bentonite–activated carbon composite beads as highly effective adsorbent for methylene blue. *Chemical Engineering Journal* **2015**, *270*, 621-630, doi:https://doi.org/10.1016/j.cej.2015.02.030.
